# Case 6/2015 - 40-Year-Old Female with Acute Chest Pain, Dyspnea and Shock

**DOI:** 10.5935/abc.20150145

**Published:** 2015-12

**Authors:** Gonçalves Daiana Zupirolli, Gutierrez Paulo Sampaio

**Affiliations:** Instituto do Coração - (InCor) - HC-FMUSP, São Paulo, SP - Brazil

**Keywords:** Aortic Dissection / surgery, Aortic Diseases / surgery, Hypertension, Marfan Syndrome

The patient is a 40-year-old female referred to the emergency unit due to thoracic pain, dyspnea and shock.

She reported a history of arterial hypertension, IgA nephropathy and HELLP syndrome in pregnancy (hypertension, hemolysis, increased liver enzymes and low platelet count).

The patient sought the Osasco Regional Hospital complaining of chest and low back pain and lower limb paresthesia (Nov 10, 2013), being then assessed and discharged. As her symptoms persisted, she returned to that hospital on the following day. On admission, the patient showed hypotension (80/50 mmHg), hyperthermia (temperatures ranging from 37.8°C to 38°C), hypoxemia, crepitant rales in both lungs, and heart murmur on auscultation.

She received empirical antibiotic therapy for pneumonia (ceftriaxone, clarithromycin and oseltamivir) and was heparinized due to suspicion of pulmonary thromboembolism.

Chest angiotomography for the diagnosis of pulmonary thromboembolism revealed type A aortic dissection, enlarged cardiac area, and areas of consolidation in the pulmonary bases.

She progressed with arterial hypotension and respiratory insufficiency due to acute pulmonary edema, requiring endotracheal intubation.

On admission at the Instituto do Coração (InCor) of the HCFMUSP, the physical examination (Nov 15, 2013) showed a sedated patient, with orotracheal intubation, FIO_2_ of 100%, O_2_ saturation of 98%, blood pressure of 90/40 mmHg, heart rate of 120 bpm, with no jugular vein stasis and asymmetrical pulses in the lower limbs. Lung auscultation revealed a decrease in respiratory sounds at the bases. Cardiac auscultation showed low heart sounds, systolic murmur +++/6+ and blowing diastolic murmur +++/6+ in the aortic region. The abdomen was flaccid, with normal gut sounds, and the liver was palpable 3 cm from the right costal margin.

Intravenous noradrenalin was introduced, and echocardiography and angiotomography of the aorta were indicated, in addition to cardiovascular surgery assessment.

The laboratory tests (Nov 15, 2013; 2h5) revealed: hemoglobin, 10.3 g/dL; red blood count, 32%; mean corpuscular volume, 97 fl; mean corpuscular hemoglobin concentration, 32 g/dL; leukocytes, 15,010/mm^3^ (neutrophils 77%, eosinophils 3%, lymphocytes 15%, monocytes 5%); platelets, 186,000/mm^3^; CK-MB, 5.49 ng/mL; troponin I, 2.43 ng/mL; urea, 51 mg/dL; creatinine, 0.97 mg/dL; magnesium, 1.6 mEq/L; C-reactive protein, 105.23 mg/L; sodium, 137 mEq/L; potassium, 4.8 mEq/L; ionized calcium, 1.17 mmol/L; arterial lactate, 12 mg/dL. Arterial blood gas analysis revealed: pH, 7.46; pCO_2_, 32.5 mm Hg; pO_2_, 129 mmHg; O_2_ saturation, 98.7%; bicarbonate, 22.9 mmol/L; base excess, (-) 0.1 mmol/L.

Echocardiography (Nov 15, 2013) showed chambers of normal measures, left ventricular ejection fraction of 65%, tricuspid aortic valve, and the following aortic diameters: root, 33 mm; and ascending aorta, 54 mm. The abdominal aorta showed intimal tear of dissection. Assessment with Doppler echocardiography and flow mapping revealed severe aortic regurgitation.

Angiotomography revealed extensive acute aortic dissection, Stanford A, initiating at the aortic root, involving the entire thoracic and abdominal aorta, and extending into the common iliac arteries, the brachiocephalic trunk and initial segment of the right common carotid artery. In addition, collapse of the true aortic lumen was observed at the end of diastole, with a suggestive image of dynamic obstruction of the left main coronary artery.

The patient was referred to emergency surgery, with orotracheal intubation and mechanical ventilation, on intravenous noradrenaline (0.5 µg/kg/min), but hypotensive. On anesthesia induction, she had cardiac arrest with pulseless electrical activity, did not respond to resuscitation maneuvers, and died (Nov 15, 2013; 15h45).

## Clinical aspects

The patient is a 40-year-old female with aortic dissection, hypovolemic shock and death. She had arterial hypertension and HELLP syndrome in pregnancy, which is characterized by hemolysis, increased liver enzymes and low platelet count.^1^

The following are considered risk factors for aortic dissection:^2^ a) arterial hypertension; b) Marfan's syndrome; c) bicuspid aortic valve; d) Turner's syndrome; e) pregnancy. In a study on the postmortem examinations performed at InCor between 1998 and 2007, 82 cases of aortic dissection were diagnosed.^3^ Most dissections were associated with other diseases, mainly arterial hypertension present in 73/82 cases (90%). Other diseases related were Marfan's syndrome, other connective tissue diseases, bicuspid aortic valve and trauma related to thoracic surgeries. Another study assessing the postmortem examination of 161 patients with aortic dissection^4^ has reported arterial hypertension in 93/161 (57.7%) cases. Other causes found were Marfan's syndrome (16 cases), atherosclerosis of the aorta (38) and degeneration of the aorta (30). Other less prevalent risk factors were bicuspid aortic valve, Turner's syndrome, aortic coarctation and pregnancy. However, the likelihood of aortic dissection due to aortic coarctation is related to the coexistence of arterial hypertension or congenital bicuspid aortic valve. Although pregnancy is considered a spontaneous risk factor for aortic dissection, that mechanism is uncommon, being 25% to 50% of the cases concomitantly associated with hypertension.

A study conducted in the state of São Paulo^5^ has reported 42,615 deaths due to aortic aneurysm and dissection over 25 years, 36,088 of which (84.7%) had the following underlying causes of death identified: aortic dissection (37%), ruptured abdominal aortic aneurysm (17.3%) and ruptured aortic aneurysm of unspecified location (17.5%). Among women, aortic dissection (38.6%) and aneurysms of unspecified location (25.4%) were reported. That study showed that among women aortic dissection was more prevalent, while among men aortic aneurysm was more frequent.

Another relevant factor in that study was the association of arterial hypertension as the cause of aortic dissection observed in 49% of the patients.

The diagnosis of aortic dissection can be difficult, because the initial signs and symptoms can be unspecific. The patient here reported sought medical care complaining of thoracic and low back pain with paresthesia of the lower limbs, and on the first clinical examination, the data obtained did not allow a diagnosis to be made. As the symptoms worsened, she returned to the hospital on the following day in a very severe condition, with hypotension, hypoxemia, fever and heart murmur.

The major classic clinical findings of a patient with aortic dissection are severe thoracic pain and syncope. A study with 464 patients^2^ has shown that severe pain of sudden onset was the most common complaint, and the clinical presentation, diverse. Syncope occurred in 12% of those patients, 10 of whom (2.2%) had no pain. Although the physical examination can provide important data for the diagnosis of aortic dissection, pulse deficit, a classical sign, was identified in less than 20% of those patients, and aortic regurgitation murmur was documented in 44% of them.

Because of its high morbidity and mortality, the diagnosis and treatment of aortic dissection should not be delayed.

The surgical treatment aims at preventing fatal complications, such as: 1) cardiac tamponade secondary to aortic rupture; 2) acute myocardial infarction; 3) symptomatic aortic insufficiency (present in the patient here reported); 4) neurological complications.^6,7^ **(Daiana Zupirolli Gonçalves, MD)**

**Diagnostic hypotheses:**
1. acute aortic dissection
2. aortic valve insufficiency
3. acute myocardial infarction

## Postmortem examination

**Figure 1:**
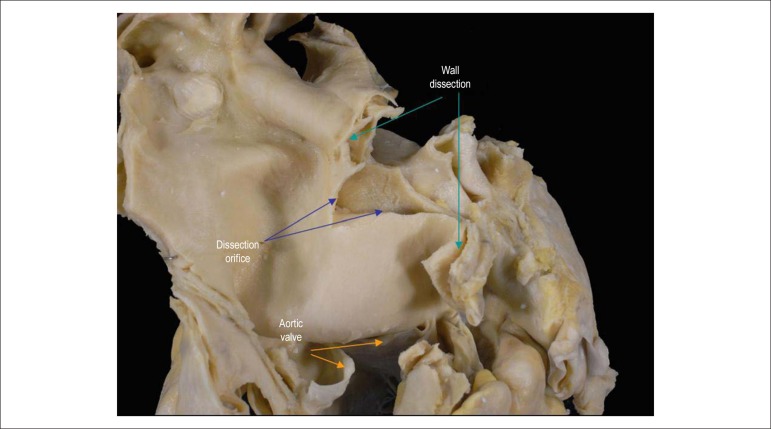
Ascending aorta showing cleavage that characterizes aortic dissection.

**Figure 2:**
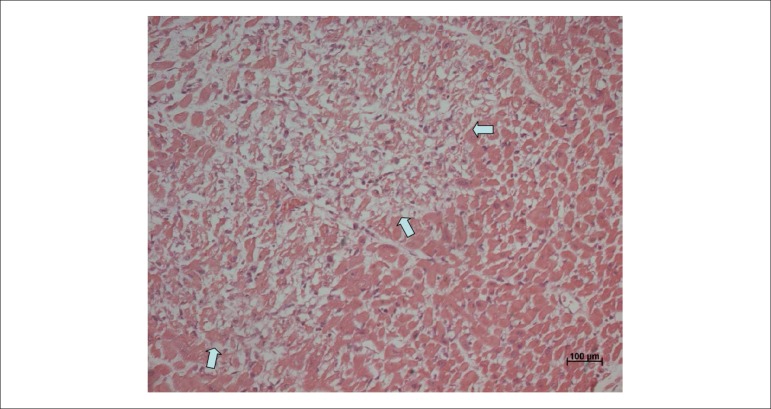
Histological section showing recent myocardial necrosis area (arrows). Hematoxylin-Eosin, 20x.

**Figure 3:**
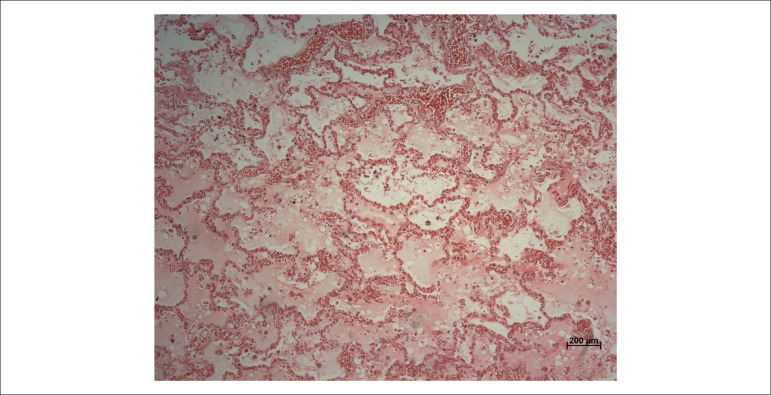
Histological section showing alveolar spaces filled with amorphous, slightly eosinophilic material, corresponding to acute pulmonary edema. Hematoxylin-Eosin, 10x.

**Figure 4:**
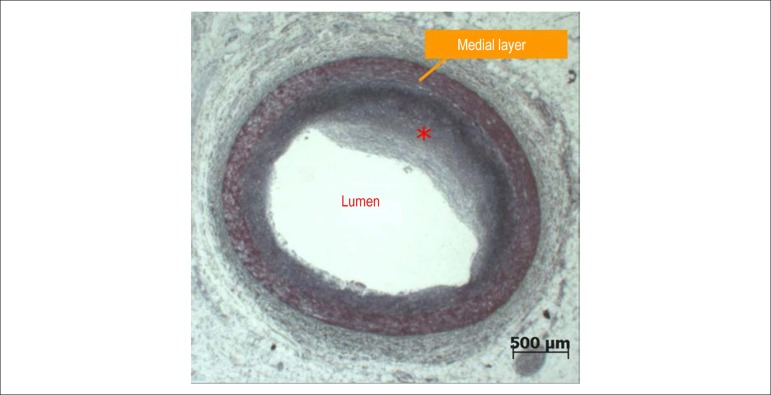
Photomicrography: cross section of the coronary artery showing only mild atherosclerosis (atherosclerotic plaque indicated by the asterisk). Movat pentachrome stain, 1x.

The postmortem examination evidenced characteristic changes of systemic arterial hypertension, such as renal arteriolosclerosis and left ventricular concentric hypertrophy. Hypertension was the underlying disease. The major disease was acute aortic dissection, with delamination extending from 1 cm above the valvar plane, site of the dissection orifice (Figure 1), to the end of the abdominal aorta. Consequent to the dissection, there were aortic insufficiency, shock leading to acute tubular necrosis and myocardial infarction with few hours of evolution (Figure 2). As a consequence of the infarction, whose size could not be precisely outlined due to the short course of disease, and of aortic insufficiency, acute pulmonary edema was observed (Figure 3), the final factor accounting for the patient's death. There was neither dissection, nor severe atherosclerosis nor thrombosis of the coronary arteries (Figure 4). **(Paulo Sampaio Gutierrez, MD)**

**Anatomopathological diagnoses:** acute aortic dissection (DeBakey type I, Stanford type A) with underlying systemic arterial hypertension

**Cause of death:** acute pulmonary edema** (Paulo Sampaio Gutierrez, MD)**

## Comments

The anatomopathological method could not precisely outline the area affected by the acute myocardial infarction, because of the short course of disease in this case. Whether the injury was large and the major cause of the pulmonary edema, or only a factor associated with aortic insufficiency, which seems less likely, could not be determined. A patient with aortic dissection can have myocardial infarction due to several mechanisms, such as hypovolemic shock, aortic dissection extending to the coronary artery, extrinsic compression of the coronary artery caused by a hematoma in the false lumen, or, as shown on this patient's angiotomography, direct reduction in the coronary flow resulting from delamination of the aorta. Despite the occurrence of myocardial infarction, acute pulmonary edema is an uncommon cause of death in patients with acute aortic dissection, which usually progresses with hypovolemia due to rupture and bleeding. Acute renal tubular necrosis can have contributed due to the increase in blood volume.

In addition, this case illustrates limits and advantages of some methods. Only *in vivo* imaging could show coronary flow obstruction, because it is a dynamic test. The postmorten examination lacks that component, and, thus, could not have confirmed that. **(Paulo Sampaio Gutierrez, MD)**

**Section Editor:** Alfredo José Mansur (ajmansur@incor.usp.br)

**Associated Editors:** Desidério Favarato(dclfavarato@incor.usp.br)

Vera Demarchi Aiello (anpvera@incor.usp.br)
